# Effects of mental practice embedded in daily therapy compared to therapy as usual in adult stroke patients in Dutch nursing homes: design of a randomised controlled trial

**DOI:** 10.1186/1471-2377-7-34

**Published:** 2007-10-15

**Authors:** Susy M Braun, Anna J Beurskens, Susanne M van Kroonenburgh, Jeroen Demarteau, Jos M Schols, Derick T Wade

**Affiliations:** 1 The centre of expertise in life sciences, Zuyd University, Heerlen, The Netherlands; 2 The department of health and technique, Zuyd University, Heerlen, The Netherlands; 3 The research centre autonomy and participation, Zuyd University, Heerlen, The Netherlands; 4 The care & public health institute, Maastricht University, Maastricht, The Netherlands; 5 Klevarie nursing home, Vivre foundation, Maastricht, The Netherlands; 6 Nursing home St. Camillus, Land van Gelre en Gulick, Roermond, The Netherlands; 7 Department of general practice, Maastricht University, The Netherlands; 8 Department Tranzo, Tilburg University, Tilburg, The Netherlands; 9 Department of rehabilitation, Maastricht University, Maastricht, The Netherlands; 10 Oxford centre for enablement, Oxford, UK

## Abstract

**Background:**

Mental practice as an additional cognitive therapy is getting increased attention in stroke rehabilitation. A systematic review shows some evidence that several techniques in which movements are rehearsed mentally might be effective but not enough to be certain. This trial investigates whether mental practice can contribute to a quicker and/or better recovery of stroke in two Dutch nursing homes. The objective is to investigate the therapeutic potential of mental practice embedded in daily therapy to improve individually chosen daily activities of adult stroke patients compared to therapy as usual. In addition, we will investigate prognostic variables and feasibility (process evaluation).

**Methods:**

A randomised, controlled, observer masked prospective trial will be conducted with adult stroke patients in the (sub)acute phase of stroke recovery. Over a six weeks intervention period the control group will receive multi professional therapy as usual. Patients in the experimental group will be instructed how to perform mental practice, and will receive care as usual in which mental practice is embedded in physical, occupation and speech therapy sessions. Outcome will be assessed at six weeks and six months. The primary outcome measure is the patient-perceived effect on performance of daily activities as assessed by an 11-point Likert Scale. Secondary outcomes are: Motricity Index, Nine Hole Peg Test, Barthel Index, Timed up and Go, 10 metres walking test, Rivermead Mobility Index. A sample size of the patients group and all therapists will be interviewed on their opinion of the experimental program to assess feasibility. All patients are asked to keep a log to determine unguided training intensity.

**Discussion:**

Advantages and disadvantages of several aspects of the chosen design are discussed.

**Trial registration:**

ISRCTN27582267

## Background

Stroke is a major health problem, which is likely to increase due to aging [1-3]. Patients are often confronted with disabilities on a physical, cognitive, social and/or communicative level. Rehabilitation of stroke patients is a time consuming process in which patients and caregivers have to learn new skills. In the Netherlands about 5,3 billion Euro are spend on rehabilitation of patients with cardiovascular diseases of which 1,5 billion is spend in nursing homes each year [4].

While it is reasonably established that the overall process of rehabilitation is effective, there is little evidence to support many specific rehabilitation therapeutic techniques [3,5]. Currently it seems that task orientated practice (i.e. practising an activity of relevance) is probably the most effective single therapeutic technique [6]. This is not dissimilar to the situation in sport where practice is the bedrock of improving skills. Indeed improving any skilled motor activity seems to depend upon continuing practice. It is perhaps this similarity that has lead to using mental practice, a technique from sports, in neurological rehabilitation.

The use of mental practice or motor imagery is well established in sports [7-12]. The principle is simple: a person imagines himself undertaking a skilled movement without actually doing the movement. It is a cognitive ability which is often used (un)consciously by all of us. Many therapists report that they use imagery in their therapy sessions already, but not systematically. They just ask the patient to imagine moving in a different way. Recently the use of mental practice has been subject to systematic research in neurological rehabilitation [13]. Although many aspects of mental practice still remain unclear, it does seem to be a promising addition to the therapy, based on best evidence. Further research into mental practice is needed, particularly where it is embedded into normal rehabilitation services.

In the Netherlands, nursing homes provide a substantial amount of stroke care although most research is restricted to academic hospitals and rehabilitation centres [14]. Consequently, more stroke research should take place in nursing homes.

The overall aim of the proposed research project is to investigate systematically the therapeutic potential of mental practice embedded in daily rehabilitation therapy on the improvement of daily activities of adult stroke patients compared to therapy as usual.

The first additional research question is which prognostic variables or patient characteristics are associated with a positive outcome in the experimental subgroup.

The second additional research question investigates the feasibility of the mental practice-based therapy as judged by the patients and therapists.

This paper reports on preliminary work, and explains the choices made in the final study design, which has been approved by the medical ethical committee of the Atrium Medical Centre & Maasland Hospital in Heerlen, The Netherlands.

### Background work

A full systematic review of previous evaluative research on mental practice was completed and has been published [13]. It showed some evidence suggesting that mental practice in stroke rehabilitation might work. However, the evidence still is weak. Furthermore, there is no consistency about or clarity on:

- the nature of the intervention itself (what was actually done?)

- the timing after stroke (when should patients be given mental practice?)

- control (what should be the control intervention?)

Consequently, we felt it important to set up a larger study. The review also highlighted several important issues to be considered in that:

- knowing what patients are doing during imagery [15],

- knowing whether patients are using mental practice [15],

- knowing what mental images of a movement the patient had [16],

- defining the core of mental practice [17],

- determining practicality in the commonest stroke rehabilitation setting (nursing homes in the Netherlands),

- determining what features predict success or failure.

These issues will be discussed also in this article.

A multi-centre, prospective, parallel-group, randomized, controlled, observer masked trial (RCT) will be conducted over a two year period (mid 2007-mid 2009) involving clinically diagnosed adult stroke patients, complying with all normal research governance procedures.

## Methods/Design

**Figure 1:**
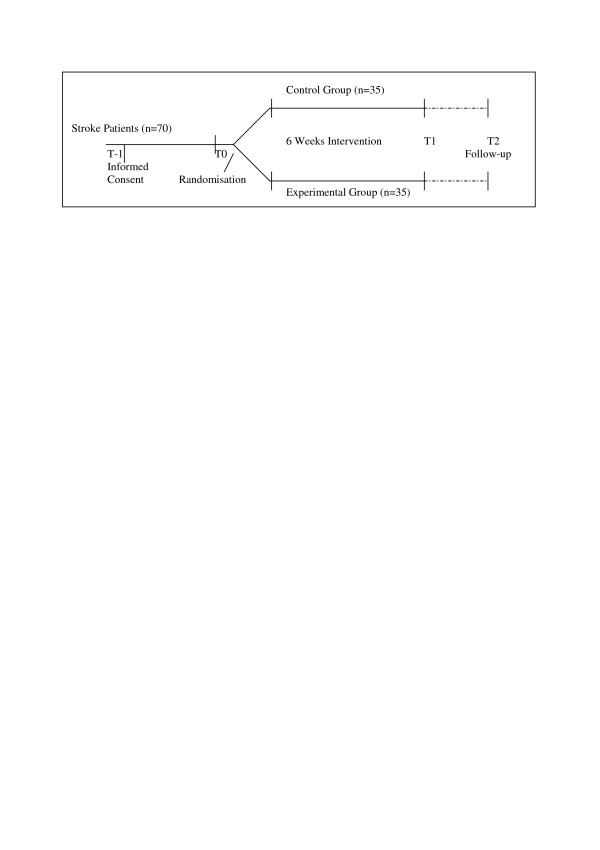
Outline of study design. T-1 = 1–4 days before baseline. T0 = baseline. T1 = 6 weeks. T2 = 6 months.

After recruitment of participants, a baseline measurement will occur (T0) followed by random assignment to either the control or experimental group. Effects of the interventions will be measured directly after the six weeks intervention period (T1) and six months after start of the intervention period (T2). Outcome measures will cover activities. Brain activity will be assessed with quantitative electroencephalography. An outline of the study design is given in figure 1.

### Study population

Seventy stroke patients will be recruited in two nursing homes by the treating physicians or other staff. There are no data available concerning ability to benefit from mental practice, and so only practical factors affect inclusion and exclusion – can the person give consent and understand the instructions?

Patients entering the RCT have to meet following inclusion criteria:

a) Clinically diagnosed adult stroke patient. In nursing homes, most of the stroke patients will be elder (> 60 years old);

b) Sufficient cognitive level and communication skills to engage in mental practice; this is a clinical judgement. Patients need to be able to follow simple instructions;

c) Between two and 10 weeks of stroke onset.

### Exclusion criteria

d) Severe additional impairments prior to stroke causing persistent disability, like rheumatic diseases, orthopaedic problems after fall.

### Sample size calculation

The calculation of the sample size is based on the primary outcome measure, perceived improvement of daily activities, like 'drinking out of a cup' and 'walking', as assessed by an 11 point Likert scale. Although arbitrary, the calculation of the sample size is based on following expectations: For us a 20% difference between the two groups in this outcome measure would be a reasonable change to aim for which means a 2.2 point change on the Numeric Rating Scale (μ1 = 2.2). There is no literature available on the standard deviation in a stroke population but in other populations the standard deviation on numerical rating scales is somewhere between 1.5 and 3.0. In this sample size calculation we will use a standard deviation of 2.25 (σ = 2.25). The power (the ability to detect a true difference in outcome) of this study is set at 90% (β = 0.10). The level of significance (likelihood of detecting a treatment effect when no effect exists) is set at 5% (α = 0.05). The power and the level of significance are generally chosen by convention.

The sample size calculation formula used for measuring the difference between two unpaired samples is: N1 = N2 = (z_1-β _+ z_1-α/2_)^2 ^* ((σ_1_^2 ^+ σ_2 _^2^)/(μ_1 _- μ_2_)^2^)

This would mean that we would need to have 19 patients in each group assuming a 50:50 random allocation. The goal of this study is to have 35 patients in each group to allow for drop-outs, loss to follow-up and uncertainty in the power calculation. A sample size of n = 70 seems realistically achievable over a 2 year period. Two nursing homes participate in this study: Klevarie nursing home (Maastricht) assesses about 200 new stroke patients each year. nursing home St. Camillus (Roermond) treats another 70 patients each year. We estimate that 40% of all treated stroke patients will be able to participate in the study, but not all will want to. If an added value of the experimental intervention can not be found in 70 patients the clinical relevance of a mental practice-based therapy should be questioned.

### Treatment of participants

All patients included in the study will receive six weeks of multi professional rehabilitation [2]. Patients in the experimental group will receive their usual therapy extended with mental practice-techniques and principles embedded in therapy sessions. Paramedical therapists will be instructed (in the theory, in workshops and in training with their patients by an external expert (SB, MK)) on how to treat the patients in the experimental group. Patients allocated to the control group can be treated by any therapist. To prevent/limit contamination in therapy of the instructed therapists, an expert (also the trainer of mental practice for the participating therapists (SB)) will monitor the contrast between the experimental and control therapy by observing therapy randomly.

### Experimental intervention

First, we would like to make some general remarks on why we chose to embed mental practice in therapy and not give mental practice as an additional intervention outside of guided practice (f.i. with an audio tape). There is some evidence that mental rehearsal should be combined regularly with the overt movement to increase imagery vividness [17-20]. Next, improving skills seems to depend on continuous practice [2,3,5,21]. Last, we believe that a higher training intensity will not only increase skills but also consolidate the mental practice technique, making the patient more confident that he/she is practicing correctly and thereby increasing compliance and motivating patients to practice unguided.

**Figure 2:**
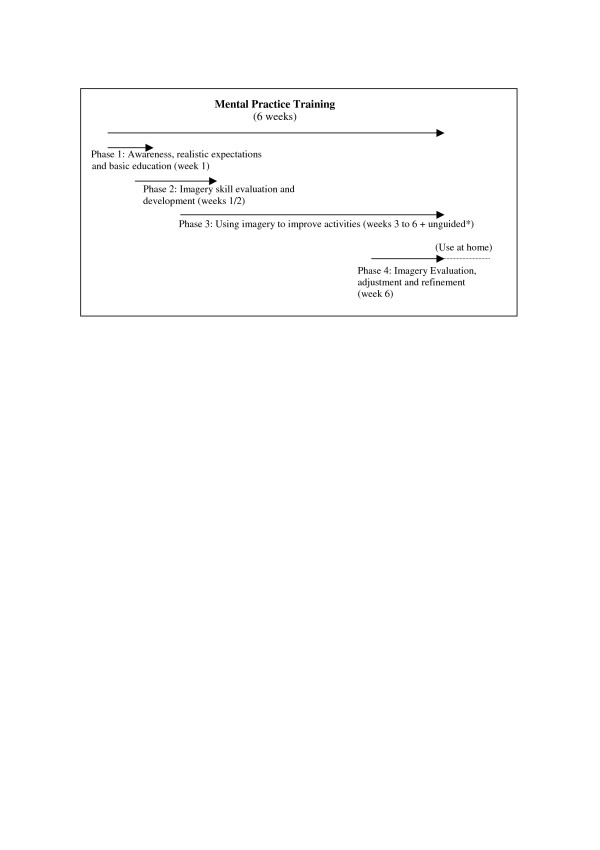
Overview of mental practice training phases and their aims. * at least 3 times 10 minutes a day. Preferably, mental practice is combined with physical or occupational therapy or the overt movement at lunch time. Time spend on mental practice unguided can be increased considerable due to compliance/motivation of the patient to practice. Training data are recorded in a personal log (diary) preferably by the patient or assisted by a member of the family or therapist/nurse.

The experimental intervention period is divided into four phases (fig. 2). In the first sessions, patients will first be familiarised with mental practice-based therapy and educated by their treating therapists as to basic imagery principles and the importance of imagery training on a regular basis (*phase 1*). There is some evidence that patients educated on and familiarised with the technique are more likely to practice in general and to practice correctly by themselves [22-24].

During the next one to two weeks, *phase 2*, they will be taught by their treating therapist how to use the mental practice technique to improve 'drinking from a cup' (occupation therapy) and/or 'walking – with(out) walking aids' (physiotherapy), depending on what they want to improve. The main reason that we chose these two activities is that patients report these activities most frequently, as activities they want to improve when starting rehabilitation. We also wanted two common activities for all patients to practice so that we can standardize the learning process and we will be able to compare results, at the end of the study. Third, 'drinking from a cup' and 'walking' are different kind of tasks; walking is repetitive with timing of different body segments (arm swing, body rotation combined with leg swing), whereas drinking is a 'forward-back movement', where the different parts of the movement seem to follow each other up in time. Perhaps these movements involve different amounts of cortical information. This could imply that certain movements are more suitable to practice mentally for they need more cortical involvement (attention) for a successful performance. Within the speech therapy, mental practice will be used to improve 'swallowing problems' or motor problems due to facial pareses, like 'smiling' symmetrically. We see no direct plausibility to improve aphasia with mental practice and have found no evidence for it in literature.

We will use results from the Structural Dimensional Analysis of Motor Memory (SDA-M) [17,18,20] to determine the basic architecture of specific goal-directed movements and thus to guide the imagery given through identifying inappropriate mental processes. It is for example used to identify weak spots in the sequence of events that should lead to a certain motor performance in sports.

We have investigated the reproducibility and feasibility of the SDA-M in a nursing home stroke population for the motor action 'drinking out of a cup'. The measuring protocol was successfully adjusted to the ability of the stroke population to process information. The measure instrument seems useful in rehabilitation [16]. The SDA-M results will be performed and interpreted by an external expert (SB) and used by the treating therapists to tailor the mental practice intervention of individual patients in the experimental group.

The vividness of imagery will be enhanced using videos of the tasks and stimulating patients to recall all sensory information during imagery, like sounds, smell, touch, pressure and taste.

The duration of the first two phases can differ depending on how fast the patient catches on to the technique. It will however not take longer than two weeks.

During the four weeks training period (*phase 3*), patients will receive guided mental practice-based therapy in their routine rehabilitation. Movement imagery will be alternated with overt movement in short individually determined blocks (for example: three times a mental rehearsal of the movement, three repetitions of the overt movement). These blocks of imagery and motor action will be interrupted by short breaks (one minute). The embedded imagery training will take place in the first 10–15 minutes of 30 minutes treating time when patients are still fresh and alert. Neural (coordination) training should take place when the nervous system is fresh (participants should not be tired due to physical exercise). Participants will be encouraged to practice unguided as much as they want. The unguided training amount is registered daily in a log. The logs are used to discuss progress in unguided therapy at the beginning of a therapy session. In a log the participants can record one week of unguided training, a new one is handed over after every week.

Three refreshment sessions can be held in which tasks are shown on a video or demonstrated. The SDA-M may be repeated if the treating therapist thinks this is useful, for example if significant change has occurred. The external expert (SB) will perform the test and again the results will be used to adjust the content of the mental practice intervention. Apart from optimizing the mental practice of 'drinking from a cup' and/or 'walking' the aim of the refreshment session is to add additional tasks (one more task per profession). This way, additional activities the patients want to improve are part of the protocol as well.

In the *fourth phase*, a general evaluation will take place to see whether any adaptations, advice or alterations are necessary in order for the patient to continue mental practice at the nursing home or at home. This will take no longer than two therapy sessions.

### Control intervention

The *control group *will receive therapy as usual in accordance with the Dutch Guidelines for Stroke Rehabilitation [2,3,21]. To compensate for the unguided imagery training, patients in the control group will be encouraged to do 'homework' as well, primarily practicing tasks that they find difficult. Participants in the control group will also be instructed to use logs.

### Study parameters/endpoints

Measurement dates are at entry in the study (T0 – baseline), after a six weeks intervention period (T1) and six months after entry (T2). Additional information during the course of rehabilitation will be collected, e.g. recurrence of stroke, other intercurrent medical problems, amount of therapy received, used medication. In addition, any deviation from the treatment protocol and any co-intervention during the six months will be recorded.

In the choice of measuring instruments following considerations were taken into account.

• The measuring instruments should have sufficient methodological quality and feasibility recorded in literature and/or be recommended by the National (Dutch) stroke Guidelines;

• If possible, the measuring instrument should be part of the standard assessment in the nursing homes to restrict the additional load on the patient;

• If the quality of a listed measuring instrument has not yet been established the measuring instruments should have evidence from other studies on their potential use in stroke rehabilitation;

• The feasibility will be measured by using semi-structured interviews (process evaluation), recording co-interventions, analysing logs and monitoring therapy content in the experimental group.

The extra load for the participants due to additional clinical testing is approximately 20 minutes at each assessment point.

### Demographics

Upon the patient's entry (T0) into the study the full neurological status and following patient characteristics will be recorded: age, gender, brain lesion site (from status), extent of hemiplegia (the perceived disability as categorised by the patient: no, mild, average and severe disability), time post stroke, ability to imagine motor acts (as perceived by the patient in four categories: no ability, blurry/unfocussed, some/parts, vivid imagery), cognitive level (MMSE), hand dominance prior to stroke. Additional information about highest educational level and sports history (no history, leisure, competition, profession) by asking the patient is recorded as well. Treating therapists are asked to predict, based on their experience, whether patients in the experimental group will benefit from a mental practice based therapy.

We will then investigate whether the two groups are comparable and whether any of these data have prognostic value.

### Main study parameter/endpoint

It is hypothesised that mental practice has the most effects on the movement that is actually mentally rehearsed [9,18,25,26]. Improvement of these activities should therefore be assessed. To measure if mental practice improves the performance of activities in the experimental group more than in the control group, an 11-point Likert scale will be used:

▪11 point Likert scale assesses the performance of the activities 'drinking' and 'walking' ranging from 10 ('excellent') to 0 ('poor') as perceived by the patient and the therapist. The physiotherapist and the occupational therapist will each score another task, which may be individually chosen and scored on the 11 point Likert scale. If the participant is treated for swallowing problems and/or motor problems due to a facial paresis by a speech therapist, he himself and the speech therapist will score motor actions within these two domains on perceived performance.

### Secondary study parameters/endpoints

• Motricity Index (MI – function (impairment) level)

The Motricity Index evaluates voluntary movement activity and the maximum muscle strength with a six point Scale in six limb movements. Reliability and Validity are sufficient in stroke populations [27].

• Barthel Index (BI – activity level)

With the Barthel Index the degree of independent performance of daily activities is measured [28]. Several versions exist. In this study an assessment form with a 20 points scale will be used [28].

• Nine Hole Peg Test (NHPT – function (activity) level)

The NHPT is a measuring instrument in which the speed of the fine hand coordination is assessed. The patient has to take nine dowels from a tray, one at a time, as fast as possible and place them in a pegboard. The time needed to complete the attempt is recorded. Only the hand that is being assessed (i.e. the affected hand) may be used. The reliability and validity are sufficient [29-31].

• Rivermead Mobility Index (RMI – activity level)

This is a staff-completed questionnaire to measure mobility disability after head injury, MS, stroke and other conditions. It comprises of 14 questions (activities scored range from turning over in bed to running) and 1 direct observation of standing for 10 seconds. Each answer is scored 'Yes' (1) or 'No' (0). The minimum score is 0 and the maximum score is 15. The higher the score is, the better the mobility.

• 10 meter walking test (TMW – activity level)

The 10-meter walking test can be used in patients able to walk independently with or without walking aids and/or orthoses. Patients should walk at a comfortable speed. The test is reliable, valid and responsive [27]. Furthermore, a significant relation between the comfortable walking speed during the TMW and the quality with which patients walk has been established [32]. Codes for not able (yet) and independent in wheelchair are 0 resp. 1.

• Timed up and go (TUG – activity level)

The TUG measures the time a patient needs to stand up from a chair, walk 3 meters at a comfortable speed, turn around, walk back and sit down. The patient is allowed to use his/her own walking aids, but no physical assistance may be given by the researcher or therapist. The test is practical and simple. The internal consistency, reliability, validity and responsiveness are sufficient [33-37].

### Optional study parameters

• QEEG (Brain-activity – neurophysiological level)

EEG activity in stroke is primarily assessed in the acute phase of stroke recovery to reveal possible epileptic activity [38]. QEEG assessment in the acute and chronic phase of recovery is not a generally performed procedure, although it might function as a reliable marker for monitoring the recovery and predicting the clinical outcomes after stroke [39,40]. In numerous studies, brain electrical activity across the sensorimotor cortex has been related to both execution and imagination of movements. The mu rhythm (8–15 Hz) seems to be connected to movement in general and is only to be found above the sensorimotor cortex [41]. In general, body movements block or suppress mu activity up to 60%, and imagination of movements generates similar suppression. It is hypothesised, that alterations in mu rhythm suppression during motor imagery in stroke would reflect distorted information processing of the sensorimotor cortex, thereby functioning as a possible marker for decreased ability of imagination of movements [42]. In the RCT, we want to investigate if the QEEG can be used as a biomarker to predict if patients are able to perform imagery and are likely to benefit from a mental practice based intervention.

In addition to the QEEG as a prognostic value, the mu suppression is used as an evaluative measure to assess progress in imagery techniques during the six weeks intervention period.

Suppression of the mu waves can be interpreted as movement related information processing. Measures of brain activity will be performed with a universal amplifier (MPAQ, Maastricht Instruments, The Netherlands) and data acquisition software (IDEEQ, Maastricht Instruments, The Netherlands). Eight sensors will be placed above the sensorimotor cortex at both hemispheres according to a standardized protocol. To ensure low skin impedance (< 5 kΩ), the skin will be cleaned with a lotion and a non-allergic gel will be used for better transmitting of the signal (Ten20 conductive gel). Results will be expressed in % of suppression of mu activity. Patients may refuse QEEG measures at T1 and T2 due to the additional load of 20 minutes per assessment. If necessary due to allergy, nickel-free electrodes will be used.

### Compliance, integrity check and feasibility of mental practice

During the mental practice intervention period, therapy compliance of the patient is monitored by using a log in which the time spent on practice is recorded on a daily basis. The diary entries will be checked by either the therapist or a member of the nursing staff. In the Dutch guidelines the amount of therapy is considered of importance with regard to effectiveness of any intervention to improve functions [2,3,21].

Furthermore, a small sample of the participants will be interviewed by the researcher on experiences and beliefs during mental practice in the experimental group and on content of therapy as usual in the control group (n = 10 in each group for both sites). These will be selected arbitrarily, and limited to those agreeing.

The therapist will be monitored as well. They will be asked to what extent they followed the given instructions from the mental practice protocol. All therapists will be interviewed on their opinion of the feasibility of the experimental intervention in every day practice. To monitor the intervention content an external expert will attend therapy sessions of participants in the experimental group unannounced. Table 1 summarises the data collection.

**Table 1:** Overview of used measures in this study

**Data**	**Time**	**Subject of assessment**
*Demographics*		
Age, gender, time post-stroke; weak side...	T0	Comparison at baseline
MMSE	T0	Comparison at baseline
Psychological assessment	T0	Comparison at baseline
Therapist' prediction on mental practice performance	T0	Comparison at baseline
*Prognostic variables*		
Amount of therapy/training	T0	Prediction on outcome
QEEG	T0	Prediction on outcome

*Primary outcome*		
11 point Likert scale: drinking and walking	T0, T1, T2	Performance on physical level 'activity'
11 point Likert scale: Patient specific tasks	T0, T1, T2	Performance on physical level 'activity'
*Secondary outcome*		
Motricity Index	T0, T1, T2	Performance on physical level 'function'
Barthel ADL scale	T0, T1, T2	Performance on physical level 'function'
Nine Hole Peg Test	T0, T1, T2	Performance on physical level 'function'
Berg Balance Scale	T0, T1, T2	Performance on physical level 'function'
Rivermead Mobility Index	T0, T1, T2	Performance on physical level 'activity'
Ten metres walk time	T0, T1, T2	Performance on physical level 'activity'
Timed up and go	T0, T1, T2	Performance on physical level 'activity'
Functional Ambulation Cat.	T0, T1, T2	Performance on physical level 'activity'
*Optional*		
QEEG	T1, T2	Performance on brain activity

*During six weeks intervention period*		
Log	Daily	
Interview	Once	A small sample (2× n = 10) will be interviewed to assess the patients opinion on feasibility of the program.
Co interventions	T1, T2	Process evaluation

*Measures with a mark are only used if required by the treating therapist (individual judgement)*.

### Randomisation, blinding and treatment allocation

#### Randomisation procedure

No stratification will take place [43]. Randomisation will take place on the participant level. Based on a computerized (block) randomisation schedule with random block size (4 or 6) seventy sequentially numbered envelopes will be prepared, with equality being achieved after every four or six. Each participant recruited will be registered and given the next sequential number, and then the envelope will be opened to determine their allocation. The randomisation procedure is the same for both sites.

### Blinding

At baseline, before randomisation the measurements will be performed by the treating therapists and psychologist of the staff. The measurements at T1 and T2 will be performed by an independent trained rater. The patients are aware of the treatment they receive, so it is not possible to blind them. The rater however will be blinded for the treatment allocation: patients will be asked by the rater not to reveal the treatment to which they were assigned. A blinding check will be performed after each of the two measurement sessions (T1 and T2). A process measure as to success of rater's blinding is the rater's opinion about the group he thinks a patient belonged to.

### Withdrawal of individual participants

Participants can leave the study at any time for any reason if they wish to do so without any consequences. If reasons are given they will be recorded. The investigator can decide to withdraw a subject from the study for urgent medical reasons. We allowed for a 20% drop out in the size calculation.

### Follow-up of participants withdrawn from treatment

The statistical analyses will be performed according to the 'intention-to-treat' principle (patients will be analysed in the treatment group to which they were randomly assigned). Patients who withdraw from treatment but allow further data collection will have data collected.

### Statistical analysis

#### Descriptive statistics

All data will be collected on paper and the records will be stored by registration number in a secure cabinet. Anonymised data will be transferred to a computer database and secured using a password. Entries in the patients' diaries and results from the interviews will be analysed qualitatively.

### Multivariate analysis

The baseline scores of the patient's demographic, primary and secondary outcomes will be used to compare the two groups. If necessary, adjustments for baseline variables will be made, using analysis of covariance. Differences at baseline and differences between the two groups on the various assessment times will be calculated. Data will be analysed using MAN(C)OVA and Generalized Estimating Equations (GEE) analyses to ascertain the effects of mental practice based therapy on different levels of outcome and to follow improvements individually in time. Regression analyses will be used to identify prognostic variables. The statistical analysis concerned with comparing the two groups will be performed according to the 'intention-to-treat' principle (patients will be analysed in the treatment group to which they were randomly assigned). Missing data will be replaced by a linear interpolation method for missing measurements. A 'last measurement carried forward' method is used to predict outcome in dropouts [44].

### Ethical considerations

Measurement instruments have been chosen as far as possible from the assessment protocol of the nursing homes, thereby minimizing the additional testing load during intake and testing for the individual patient. Table 2 gives an overview of the main ethical considerations.

**Table 2:** Overview of the ethical consideration

**Question**	**Comment**
How great was the change in clinical practice?	Minor: use of consistent advice and consistent technique in both groups (embedded in therapy as usual)
What extra burden was imposed upon the patient(s)?	Moderate: some time in collecting data from measures not standard in the protocols of the nursing homes.
What additional risks did the patient(s) (or other participants) face?	Minor: from the mental practice intervention none can be thought of at present, results of QEEG could generate some ethical issues for the researcher if major unexpected abnormalities are discovered
What benefit might accrue to the patient (or other participants)?	Moderate: experimental treatment may be of complementary value to current practice
What benefit might accrue to Society?	Moderate: the study should detect any clinically relevant difference in treatment. Papers in peer reviewed journals will be submitted and researchers will learn and teach in research methodology
Was each participant informed about the study and able to choose whether or not to participate?	The patient is informed orally and in writing. Participation in the study may be considered for at least 2 days. Patient may withdraw from the study at any time without giving reason why. This will not affect treatment negatively
Was the method of recruiting participants fair and appropriate?	As little is known about specific selection of stroke patients likely to benefit most from a mental practice regime in rehabilitation, inclusion criteria were kept as broad as possible

### Recruitment and consent

The method of recruitment should be fair, neither disadvantaging some patients nor advantaging others. All patients will received full information and will be given at least 48 hours to decide whether they wish to participate in the research, and will be able to withdraw at any time without affecting their other rehabilitation. Witnessed consent will be obtained. Patients may contact an independent physician for information and advice. The patient is free to refuse participation.

### Benefits and risks assessment, group relatedness

As there are no invasive interventions, nor any untested experimental measurement instruments used, there is no additional risk associated with the additional assessments or treatment of the patient.

There will be a small extra burden potentially placed on patients. They will spend more time seeing the researcher. They will be asked to answer questions, fill in questionnaires, and sometimes to undertake activities or tasks that might be timed. However none of this will be especially uncomfortable or troublesome, and all can be undertaken by the patient at their own pace.

The risks faced by patients are no greater than those risks they face during routine rehabilitation practice. The only potential exception to this is the QEEG. A QEEG in itself carries no direct risks. Theoretically, a patient might be allergic to the materials used, but each patient will be asked if they are allergic to these materials (e.g. the metal involved in the electrode) and a anti-allergic gel will be used to improve signal measures.

## Discussion

This study poses several interesting problems when considering its design. When we started out deciding how the trial should be performed, we first discussed the goals of our research. We thought it important that mental practice should be applicable to as many stroke patients and usable in as many settings as possible. This meant that the costs for the program should be low, therapists should be instructed easily and the content of mental practice should not be difficult to follow, so that not too many exclusion criteria should be set. We then decided to run the study in nursing homes, because they have a major role in stroke rehabilitation in The Netherlands [14]. The choice for this setting has many implications for the content of the experimental intervention and the recruitment of eligible participants. Two important aspects of recruitment concern the selection criteria and the best time to approach the patients. Our third main concern was to define the control intervention and the outcome measures in order to identify effects of mental practice on stroke recovery. These aspects will be discussed below.

### Strategy vs tactics: teach how to do mental practice in general, or teach only a specific task?

Although all nursing homes work according to the best available evidence, there are considerable differences in tasks and organisation of the nursing homes, depending for instance if they are part of specific stroke services or not. This reflects in variations in time post stroke at submission, the neurological assessments, tasks of professionals and routeing of the patient during the rehabilitation process and also in the duration of the rehabilitation process. We wanted to create an intervention protocol that could be used in any nursing home, independent of the organisation form.

Second, we wanted therapists of different disciplines to use mental practice in motor learning tasks of the same patient, so that they could reinforce each other's therapy.

We therefore made the explicit choice to only set a fixed theoretical framework for using mental practice, but also leave room to tailor therapy to the preferences and abilities of the patient, thereby giving the professional space to use his/her experience (real practice) instead of forcing patients into a fixed intervention program.

### Incorporation into routine working practice

In reported studies, sometimes the physiotherapist, the occupational therapist or the psychologist instructed and guided the patients through the mental practice and sometimes an audio tape was used for this purpose [13]. There is no evidence that a certain caregiver is a better mental practice coach than another. As stated before we chose to incorporate mental practice into every paramedical therapy session. To ensure that all treating therapists of the experimental group were informed of what the other therapists were doing and to enhance and use imagery skills of a patient, all physiotherapy, speech therapy and occupational therapy sessions were recorded in one central log. If time does not allow the therapists to discuss patients in person, they are still able to see what others have done and use this information for their own sessions.

In order to use their expertise for tailoring mental practice, a therapist should have practical experience in teaching and monitoring it in daily routine. Therapists need to be instructed thoroughly, not only theoretically but especially with regard to 'hands on' skills. Therefore, a great part of the instruction needs to consist of workshops (showing and giving examples) and training on the job (guiding). Sufficient time needs to be reserved for therapists to get familiar with mental practice and teaching it confidently. We incorporated a preliminary phase of about five months for the therapists to get comfortable with mental practice, gaining experience and incorporating it into routine working practice, before starting the trial.

Because we randomised on the patient level (and not on f.i. institute or therapist level) and because we trained all therapists involved in the trial (giving both the experimental and control intervention), we faced a challenge: avoiding possible contamination of mental practice into the control content. We choose for randomisation on patient level because we felt that real care of patients should come first (see: *selecting patients*). We trained all therapists to maximize recruitment within the nursing homes. Both therapists and patients can diminish the contrast between the experimental and control intervention.

Caregivers who were made enthusiastic about a new approach and have just spend a lot of time learning how to use mental practice as an additional tool may be tempted to use it as much as possible, whenever possible. This could decrease the differences between the two compared interventions. There is some evidence that altering professional behaviour is not very easy [45]. Therapists will probably need more effort to consciously implement mental practice into their daily routines then copying them in the control group. Nonetheless, therapists will undergo an integrity check too, determining how well they were able to distinguish between the two interventions in therapy. During an interview, therapists will be asked to which extend they were able to stick to the intervention contents (subjective opinion). Furthermore, the external expert will attend therapy sessions of all therapists unannounced and at random throughout the two year trial period. With these measures, we will try to maximize the contrast between the compared interventions. In case contamination does take place, we will report it as fair as possible.

From the patient's point of view, we believe there is a big difference between telling patients to imagine movements and actually teaching it. It is more probable that patients that were taught will successfully use mental practice on a regular basis. A big part of the effect will probably come from the practice outside of therapy, unguided.

### Selecting patients

Ethical considerations in recruiting patients should always be taken in account, especially if so little is known about effects and prognostic values, as is the case in imagery research. Because we do not know whether mental practice will lead to better and/or quicker recover in this research the only 'ethical' design seems to be a randomised trial. Data analysis with GEE will give us more ideas on improvements on an individual basis.

Patients unable to learn imagery should not be frustrated unnecessary, but we also do not want to withhold mental practice from patients who might well benefit from mental practice. Since we do not know who might benefit from mental practice we believe it ethical to include anyone able and willing to participate in the research. Within this study, the participation judgement is made based on the extend of attention a patient has, as well as the amount of working memory he/she has and the ability to perceive different information. Participants need to be able to think practical but analytical about a movement. Predictions of the treating therapists on the ability of the patient to participate in the study will be discussed during multi disciplinary meetings of the paramedical team and a prediction of the patient's ability to participate in mental practice will be made based on consensus.

We are aware that patients who just have had a stroke are extremely vulnerable and should be given time to cope with their new situation. So when should recruitment start? In preliminary work, we found that about 25% of the acute stroke patients (one to two weeks post stroke) in the nursing homes were willing to participate in a pilot study. We decided to start recruitment within the RCT at any time between two and ten weeks post stroke which should maximise recruitment while maintaining the focus on patients in the acute phase. Those patients who are recovering well and able to start earlier will probably start at two-three weeks but patients who are sicker or slower in recovering still have an opportunity to start up to ten weeks. This approach should, oddly, reduce heterogeneity in the population studied because each person will be recruited at a similar stage in their own recovery trajectory.

### Knowing if people are practicing

Two aspects are important with regard to compliance of the patient to the experimental intervention. 1. Are patients actually thinking about movements during mental practice and 2. Are they reporting the unguided therapy time correctly?

At this point, we do not know of any measure that can measure thoughts. In different studies, the main way to check if participants are imaging movements is the indirect way of interviewing. Our experience is that one can get a good idea of what the patient has been doing, just by asking. We therefore chose to use semi-structured interviews in our trial to check on what patients are doing during imagery. However, we also wanted a quantitative measure to verify self-reports. The idea of using QEEG as a biomarker for imagery came from a study Ramachandran performed with autistic children [46]. Mu wave suppression seems to be a key factor in determining whether a participant is imagining movement or not. In the trial, we want to assess if mu-wave suppression during imagery is trainable and whether it can be used as a prognostic value in the nursing home stroke population.

One of the benefits of mental practice is that it can increase the therapy amount considerably; patients are not dependent on time spend in the gym with therapists, but can exercise safely any time, anywhere [22,23,47-56]. This results in giving the patient more autonomy over his rehabilitation process, which might well motivate certain participants to practice.

Therapy amount could well be a prognostic variable for outcome. It is therefore important to register unguided therapy time as detailed as possible. A log was developed for this purpose. In it, the patient can describe on a daily basis, what activity he/she has practiced mentally, for how long and how well it went. Additionally the patient can score how he/she felt that day by marking one of three smiley's (bad day, neutral day, good day). At the bottom of each page (day), space is left open for comments and remarks, for instance to report if anything unusual had happened. To encourage patients to use the log, every session starts with looking at the log's entries and discussing them with the patient. By involving family members and the Nursing staff, we hope to come as near as possible to the actually practiced time.

### Control for mental practice training

By embedding mental practice in the regular therapy sessions and therapy time, we tried to minimize the additional training time in the experimental group. About four to six additional hours are made during the six weeks intervention time (one hour per week). The majority of these six hours are spend on performing the SDA-M in the second phase of the mental practice protocol (three-four hours) during regular therapy time. The external expert will perform this assessment and discuss outcome with the treating therapists. The other two to three hours are used instructing participants on what mental practice is, tailoring the intervention to the patient's abilities and preferences and explaining the log as well as interviewing a part of the patient group.

We decided to compare mental practice embedded in therapy with therapy as usual (TAU) and compensate for extra time spend with patients and given attention were possible. The SDA-M is not performed in the control group. During these hours, participants from the control group will have therapy as usual.

To compensate for attention, participants from the control group will be instructed to keep a log on their homework (movements) and then score the actual performance of these actions. Within the control group an equally big sub group will be interviewed on their perceptions and believes with regard to the control intervention.

### Outcome measurement

There is little known about what specific aspect or combinations of aspects of mental practice lead to positive results [18,19]. Outcome measures within neurological studies are often chosen to assess physical improvement, but imagery can also have effects on motivation and altering cognitions and feelings, like anxiety. Martin and co-workers developed an applied model, based on models from cognitive psychology, to provide a guiding framework that describes how an athlete can use imagery to achieve a variety of cognitive, affective, and behavioral changes across different sport situations [17]. It is very important that such a (for neurological rehabilitation) adjusted model were applied in neurological rehabilitation as well, to ensure future comparison of studies.

We specifically chose to use mental practice to improve physical performance, trying to leave mental practice content that has effects on other domains, out of the intervention protocol. The measuring instruments were chosen with this information in mind. We thought it very important for patients to be able to choose the tasks they wanted to improve, for they should be relevant for the client. Just as in athletes, patients then use mental practice for specific motor actions. Exactly these movements should then improve. The chosen tasks, apart from 'drinking from a cup' and/or 'walking', are likely to vary greatly, since they depend on the patient's preferences. The 11 point Likert Scale was chosen as the primary measure because it seems most plausible that effects of mental practice should first and mainly be detectable on the rehearsed tasks. Second, we wanted one measure that could be used for any kind of motor action. Subjective changes in the performance of the task from both the therapist's view as the patient's view seem equally important. The secondary measures were chosen to reflect on as many tasks as possible.

### Final remark

By describing the design, we hope to contribute to the discussion on how mental practice should be introduced in stroke rehabilitation. Results from this study, might clarify some aspects of mental practice in stroke rehabilitation and give further leads on how to do it, or how not to.

## Competing interests

The author(s) declare that they have no competing interests.

## Authors' contributions

SB, AB and DW participated in the design of the trial. SB, SvK, JD and JS participated in the coordination, try out and fine tuning prior to the study. JD and SK are the leading researches at the trial sites, SB the coordinating researcher. All authors read and approved the final manuscript.

## Pre-publication history

The pre-publication history for this paper can be accessed here:


